# Brand interchangeability of pepsinogen tests in the real-world setting after eradication of *Helicobacter pylori*: a community-based study

**DOI:** 10.1186/s12876-022-02155-7

**Published:** 2022-02-18

**Authors:** Tsung-Hsien Chiang, Yen-Nien Chen, Yi-Ru Chen, Yu-Hua Tseng, Chun-Fu Shieh, Cheng-Ying Liu, Han-Mo Chiu, Hung Chiang, Chia-Tung Shun, Ming-Shiang Wu, Jaw-Town Lin, Yi-Chia Lee

**Affiliations:** 1 Department of Internal Medicine, College of Medicine, National Taiwan University, Taipei, Taiwan; 2 Department of Integrated Diagnostics and Therapeutics, National Taiwan University Hospital, Taipei, Taiwan; 3 Department of Medicine, National Taiwan University Cancer Center, Taipei, Taiwan; 4 Department of Health Care Management, National Taipei University of Nursing and Health Sciences, Taipei, Taiwan; 5 Lienchiang County Public Health Bureau, Nangan Township, Lienchiang County, Matsu Taiwan; 6 Lienchiang County Hospital, Nangan Township, Lienchiang County, Matsu Taiwan; 7 Lienchiang County Government, Nangan Township, Lienchiang County, Matsu Taiwan; 8 Graduate Institute of Epidemiology and Preventive Medicine, College of Public Health, National Taiwan University, Taipei, Taiwan; 9 Taipei Institute of Pathology, Taipei, Taiwan; 10 Department and Graduate Institute of Forensic Medicine, College of Medicine, National Taiwan University, Taipei, Taiwan; 11 Division of Gastroenterology and Hepatology, Department of Internal Medicine, E-Da Hospital, Kaohsiung, Taiwan; 12 Department of Medical Research, National Taiwan University Hospital, Taipei, Taiwan

**Keywords:** *Helicobacter pylori*, Pepsinogen, Screening, Positive predictive value, Detection rate

## Abstract

**Background:**

Serum pepsinogen (PG) is recommended as a screening test for premalignant gastric lesions. However, real-world evidence demonstrating its applicability and equivalence between different test brands is limited.

**Methods:**

Mass screening began in 2018 in a high-risk Taiwanese population after eradication of *Helicobacter pylori*, with the first stage of two PG tests (GastroPanel^®^, Helsinki, Finland and LZ-Test^®^, Tokyo, Japan) and the second stage of endoscopy. A positive test was defined as PG-I < 30 ng/mL or PG-I/II ratio < 3 for GastroPanel^®^ and PG-I ≤ 70 ng/mL and PG-I/II ratio ≤ 3 for LZ-Test^®^. Index lesions included atrophic gastritis and intestinal metaplasia. Test performance was evaluated based on the participation rate, positivity rate, referral rate, positive predictive value (PPV), and the detection rate.

**Results:**

Among 7616 eligible participants, 5117 (67.2%) received PG tests and 284 (5.6%) tested positive. Of those who tested positive, 105 (37.0%) underwent endoscopy. Overall PPVs for atrophic gastritis and intestinal metaplasia were 12.4% and 18.9%, respectively, with detection rates of 2.5 and 3.9 per 1000, respectively. Correlations of numerical measures between tests were high and the agreements of test results were substantial. The PPVs (16.3% vs. 16.3% and 23.8% vs. 21.3%, *P* = 1.00 and 0.71, respectively), detection rates (2.5 vs. 2.5 and 3.7 vs. 3.3 per 1000, *P* = 1.00 and 0.27, respectively), and the stage distributions of gastritis were all comparable, which were confirmed by multiple regression analyses.

**Conclusions:**

PG testing is effective for mass screening after eradication of *H. pylori*. Tests from different manufacturers, even using different analytical methods and cutoff criteria, can perform equivalently.

**Supplementary Information:**

The online version contains supplementary material available at 10.1186/s12876-022-02155-7.

## Introduction

Gastric cancer is the sixth most common cancer and the third most common cause of cancer deaths worldwide [1]. Screening and prevention strategies are urgently needed to reduce the significant burden of gastric cancer. *Helicobacter pylori* infection is the initiator for gastric cancer carcinogenesis [2], so active screening and eradication of this bacterium is considered one of the best strategies [3]. However, the benefit of such an approach depends on the magnitude of pre-existing molecular damages in the gastric mucosae [4]. For patients who already harbor premalignant gastric lesions after long-term inflammation, such as atrophic gastritis and intestinal metaplasia, they can retain irreversible changes after the eradication treatment [5, 6]. Therefore, when *H. pylori* eradication is increasingly adopted as a healthcare policy, a non-invasive test is needed to accurately identify candidates most likely to benefit from endoscopy, in order to allocate the limited resources.

Serum pepsinogen (PG), a proenzyme of pepsin secreted from gastric mucosae, is released into the systematic circulation and thus becomes measurable, making it a good choice [7]. PG consists mainly of two subtypes: PG-I and PG-II; the former is secreted by the fundic glands and the latter is secreted by the pyloric and Brunner’s glands [8]. When long standing *H. pylori* infection leads to the loss of secretory glands, both PG-I and PG-II will decline, providing quantitative measures for the presence and severity of mucosal damages [9, 10]. However, some barriers exist to its clinical application. First, PG testing isn’t routinely used as a tool for mass screening, so real-world evidence of its effectiveness, in terms of population coverage, acceptance among patients, and diagnostic accuracy, is limited. Second, in the current free-market system, different brands of PG testing may be chosen. Although results are generated from the same antibody-antigen reaction, their methods to measure the concentration of antibody-antigen complexes differ. This may include colorimetric methods, radioimmunoassay, enzyme-linked immunosorbent assay (ELISA), and chemiluminescence [11–13]. As a result, the numerical measures and the cutoff criteria for a positive test are fraught with significant variations, leading to uncertainty about the interchangeability of results. Third, after *H. pylori* eradication, the back diffusion of PG-I and PG-II from stomach to the systematic circulation is reduced because the mucosal integrity is improved through the attenuation of mucosal inflammation [14]. However, our understanding of the performance of PG tests under these circumstances is limited.

In Taiwan, gastric cancer preventative programs have been implemented, allowing us a large enough high-risk cohort to study these questions [3, 14]. We therefore used this cohort to evaluate the performance of PG testing in a post-eradication population as well as determine if two different brands of PG testing, with different measurement methods and cutoff criteria, would perform equivalently in the prediction of the premalignant gastric lesions.

## Methods

### Study population

Our eligible population comprised 7616 inhabitants of an offshore archipelago (Matsu Islands, Taiwan), who were aged 30 years or more and registered in the population registry. Because of the high gastric cancer incidence in this population, a mass screening and eradication program of *H. pylori* has been implemented since 2004 [3]. All eligible inhabitants were invited by mail, telephone, or announcement in social media and newspapers to attend the screening program. Participants’ demographic data, social habits, and medical histories were recorded in a structured questionnaire. *H. pylori* infection was determined using the ^13^C-urea breath test, those testing positive received eradication treatment, and those who failed initial treatment were retreated [15, 16]. By the end of 2018, six rounds of mass eradication have been conducted. As a result, the prevalence rate of *H. pylori* infection in the Matsu Islands declined from 64.2% before the mass eradication program to about 10% recently, accompanied by a 53% reduction in gastric cancer cases [14].

### Study design

Since scattered cases of gastric cancer occurred during the mass eradication program, a two-stage endoscopic screening program was added to the chemoprevention program in 2015, with the main purpose to identify participants who were still at high risk for gastric cancer after *H. pylori* eradication and provide them with endoscopic screening. This program was done using the serological test with PG, in which the test positives would be referred for endoscopic examination and histological evaluation. Preliminary results were reported previously [17].

In 2018, because various brands of PG were available in the market, we tested whether or not the performance of two brands, the GastroPanel^®^ (Biohit HealthCare, Helsinki, Finland) and the LZ-Test^®^ (Eiken Chemical Co., Ltd, Tokyo, Japan), was equivalent. We applied a parallel test design [18], in which participants with positive PG results for either one of the two tests would be referred for endoscopic examination. We assumed the possibility of false negatives could be lower than those based on a single test.

These tests also included measurements of the anti-*H. pylori* IgG or gastrin-17; however, since our initial experience indicated that the values of these two measures had been reduced after *H. pylori* eradication [17], we only analyzed the results of the PG-I, PG-II, and PG-I/II ratio in this study. The study protocol was approved by the Institutional. Review Board (IRB) of National Taiwan University Hospital (IRB No: 201406021RINA), and written informed consent was obtained from all participants before they entered the study.

### Serological tests

After an overnight fast, participants received blood sampling for both GastroPanel^®^ and LZ-Test^®^ at the same time. Participants were requested to stop taking acid suppressing drugs, particularly proton-pump inhibitors, at least one week before testing. After the blood sample was taken by venipuncture, it was placed into the serum tube without any additives. The tube was placed in an upright position for at least 30 min at room temperature to allow for blood clot formation. Then, after the blood sample was centrifuged for 10 min, it was stored at – 20 ℃ and prepared for measurements of PG.

The GastroPanel^®^ test applied the ELISA method to measure the serum concentration of antigen–antibody complexes based on color intensity. The PG-specific antibodies were coated onto the surface of a microplate. The blood sample was then added to the wells, creating the antibody-antigen reaction. A secondary antibody linked to an enzyme was used to bind the antigen–antibody complexes. Finally, color-producing substrates were added to react with the enzyme and color was produced. The PG concentration in the serum sample could be quantified according to the intensity of color. The cutoff concentrations recommended by the manufacturer were set at PG-I < 30 ng/mL or PG-I/II ratio < 3 [19]. The sensitivity, specificity, and positive likelihood ratio in the prediction of premalignant gastric lesions had been reported as about 40%, 93%, and 6.0, respectively [20].

The LZ-Test^®^ applied the latex-enhanced turbidimetric immunoassay (L-TIA) that measured the concentration of antigen–antibody complexes based on the optical turbidity. The latex reagent was prepared through the binding between the PG-specific antibodies and the latex particles. After the latex particles reacted with the PG presented in the blood samples, the agglutination reaction would change the turbidity, which could be therefore quantified. The cutoff concentrations recommended by the manufacturer were set at PG-I ≤ 70 ng/mL and PG-I/II ratio ≤ 3 [21]. The sensitivity, specificity, and positive likelihood ratio in the prediction of premalignant gastric lesions had been reported as about 59%, 89%, and 5.5, respectively [22].

### Endoscopic and histological evaluations

During the endoscopic examination, biopsy specimens were taken from gastric mucosa in the antrum (from the greater and lesser curvatures 2–3 cm from the pylorus) and corpus (one each from the lesser and greater curvature at the middle corpus) according to the modified Sydney protocol [23]. Sampling was done at the same locations in all participants to maintain consistency. All specimens were fixed in formalin for histological evaluation. A senior histopathologist (Dr. H Chiang), blinded to participants’ clinical status and test results, performed all histological assessments. Specimens were graded, using the Updated Sydney Classification, as acute inflammation (polymorphonuclear infiltrates), chronic inflammation (lymphoplasmacytic infiltrates), atrophic gastritis (loss of glandular tissue and fibrous replacement), or intestinal metaplasia (presence of goblet cells and absorptive cells) [23]; the severity of each category was rated as none, mild, moderate, or marked. And the severity of premalignant lesions were classified using the criteria of Operative Link for Gastritis Assessment of Atrophic Gastritis (OLGA) and Operative Link for Gastritis Assessment of Intestinal Metaplasia (OLGIM) [24, 25].

### Statistical analysis

For the baseline characteristics, categorical data were expressed as a percentage and continuous data were expressed as a mean, with standard deviation. The baseline characteristics of participants were compared using the Student *t*-test or chi-square test. The performance of the whole program was evaluated according to the consecutive steps of an organized screening program, including the invitation, participation, testing, referral, and the final diagnosis. The indicators included the test positivity rates (the number of positive cases divided by the number of participants), referral rate for endoscopic examinations (the number of endoscopies divided by the number of positive cases), positive predictive value (PPV: the number of participants diagnosed with premalignant gastric lesions divided by the number of diagnostic endoscopies), and the detection rate (the number participants diagnosed with premalignant gastric lesions divided by the number of participants).

Two brands of PG tests were then compared. First, the Pearson’s correlation coefficient (a level of 0.7 or more indicated a strong correlation) was applied for the numerical measures of PG-I, PG-II, and PG-I/II ratio, and the Kappa statistics (a level of 0.6 or more indicated substantial agreement) was applied for the positivity rates. Second, the positivity rate, referral rate, PPV, and detection rate were compared between two tests using the two sample proportional test. To adjust for differences in baseline characteristics, logistic regression analyses were performed, with the results expressed as the crude and adjusted odds ratios and the corresponding 95% confidence intervals (CIs). Third, the positivity rates between two tests in patients with premalignant lesions were compared using the two-sample proportion test according to the OLGA and OLGIM stages.

All statistical analyses were performed using Stata 14 software (StataCorp LLC, College Station, TX, USA). All *P*-values were two-sided, with *P* < 0.05 considered statistically significant.

## Results

### Baseline characteristics

**Fig. 1:**
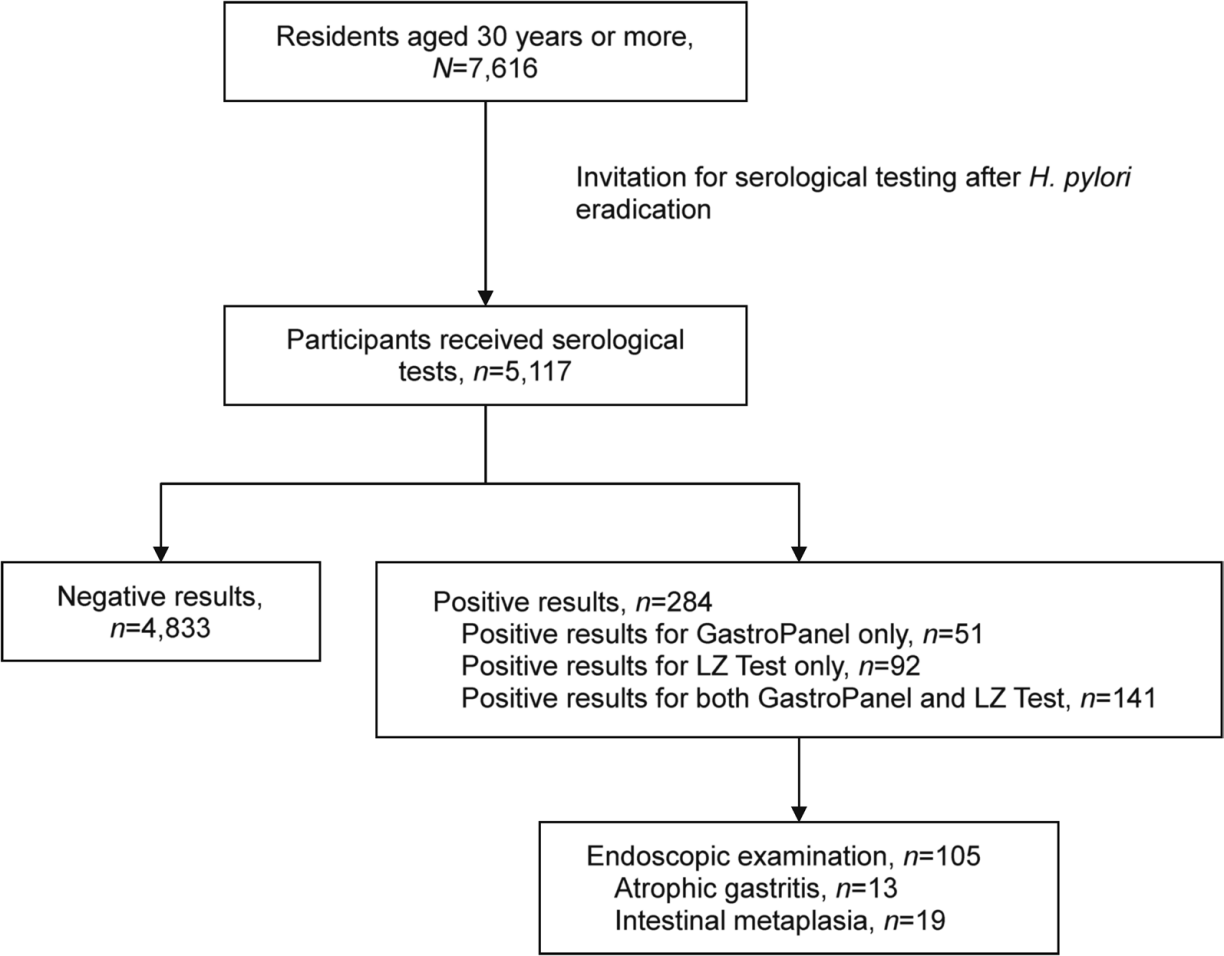
Study flowchart

The study flowchart is shown in Fig. 1. Among 7616 eligible participants, 5117 received the PG tests, leading to a coverage rate of 67.2%. Among these participants, 284 (5.6%) had positive results. Among participants tested positive, 51 participants tested positive for GastroPanel^®^ only, 92 tested positive for LZ-Test^®^ only, and 141 tested positive for both tests. A total of 105 (37%) participants tested positive and underwent endoscopic examinations: 13 were diagnosed with atrophic gastritis, 19 diagnosed with intestinal metaplasia, and 7 diagnosed with both atrophic gastritis and intestinal metaplasia.

Baseline characteristics of participants are shown in Table 1. Participants who tested positive for either GastroPanel^®^ or LZ-Test^®^ were older than those who tested negative (58.1 vs. 54.8 years, *P* < 0.001). A history of hypertension was more common in those who tested positive than those who tested negative (38.7% vs. 29.9%, *P* = 0.002), which was probably because the subjects who tested positive were older than those who tested negative. The mean value of body mass index was similar between those who tested positive and negative. Also, the proportion of male sex, cigarette smokers, alcohol drinkers, betel nut chewers, and other medical conditions, including diabetes mellitus, hyperlipidemia, cardiovascular disease, stroke, chronic hepatitis B, chronic hepatitis C, and chronic kidney disease, were all comparable between participants with positive and negative results.

**Table 1:** Baseline characteristics of the screening participants stratified by the results of the PG tests

Screening participants	Participants (*n* = 5117)	Negative result (*n* = 4833)	Positive result* (*n* = 284)	*P* value^†^
Mean age, years (SD; range)	55.0 (12.9; 30–100)	54.8 (12.9; 30–100)	58.1 (13.5; 30–96)	< 0.001
Male sex, no. (%)	2510 (49.1)	2364 (48.4)	146 (51.4)	0.33
Body mass index, kg/m^2^ (SD; range)	25.1 (4.0; 14.6–52.1)	25.1 (4.0; 14.6–52.1)	25.1 (3.9; 15.8–37.5)	0.93
Social habits, no. (%)				
Current smoker	752 (14.7)	708 (14.6)	44 (15.5)	0.68
Regular alcohol drinking	320 (6.3)	307 (6.3)	13 (4.6)	0.25
Betel nut chewing	221 (4.3)	208 (4.3)	13 (4.6)	0.81
Medical history, no. (%)				
Hypertension	1553 (30.3)	1443 (29.9)	110 (38.7)	0.002
Diabetes mellitus	464 (9.1)	441 (9.1)	23 (8.1)	0.57
Hyperlipidemia	375 (7.3)	346 (7.2)	29 (10.2)	0.06
Cardiovascular disease	269 (5.3)	253 (5.2)	16 (5.6)	0.77
Stroke	30 (0.6)	29 (0.6)	1 (0.4)	0.67
Chronic hepatitis B	626 (12.2)	586 (12.1)	40 (14.1)	0.32
Chronic hepatitis C	27 (0.5)	24 (0.5)	3 (1.1)	0.18
Chronic kidney disease	56 (1.1)	54 (1.1)	2 (0.7)	0.53

*SD* standard deviation

^*^Test positives for either GastroPanel (Biohit HealthCare, Helsinki, Finland) or LZ-Test (Eiken Chemical Co., Ltd, Tokyo, Japan)

^†^*P* < 0.05 in the comparison between the test positives and negatives

The comparison of baseline data between endoscopic receivers and refusers is shown in Additional file 1: Table S1. No significant differences were noted except that the proportion of hyperlipidemia and cardiovascular disease were higher in the endoscopic receivers.

### Performance of the whole program

The number of positive tests, positivity rates, number of endoscopies, and the referral rate of endoscopic examinations, which are stratified by age, sex, and testing brand, are shown in Table 2. The positivity rates were 3.8%, 4.6%, and 5.6% for GastroPanel^®^, LZ-Test^®^, and the parallel combination (i.e. either one was positive), respectively. The positivity rate of the parallel combination was higher than that of GastroPanel^®^ (5.6% vs. 3.8%, *P* < 0.001) and LZ-Test^®^ (5.6% vs. 4.6%, *P* = 0.021). The positivity rates of the parallel combination were slightly higher for males (5.8% vs. 5.3%, *P* = 0.43), but were significantly higher in the older-age group (≥ 50 years) than the younger-age group (30–49 years) (6.1% vs. 4.5%, *P* = 0.016).

**Table 2:** The process indicators of screening, stratified by the age, gender, and the brands of PG testing

Brands of PG test	Participants, no	Positive test, no			Positivity rate (%)			Endoscopic examination, no			Referral rate for endoscopic examination (%)		
		Test 1	Test 2	Test 1 + 2	Test 1	Test 2	Test 1 + 2	Test 1	Test 2	Test 1 + 2	Test 1	Test 2	Test 1 + 2
Male													
30–49 years	842	17	29	37	2.0	3.4	4.4	10	9	15	58.8	31.0	40.5
≥ 50 years	1668	85	93	109	5.1	5.6	6.5	34	36	43	40.0	38.7	39.4
Subtotal	2510	102	122	146	4.1	4.9	5.8	44	45	58	43.1	36.9	39.7
Female													
30–49 years	1006	24	36	46	2.4	3.6	4.6	8	8	12	33.3	22.2	26.1
≥ 50 years	1601	66	75	92	4.1	4.7	5.7	27	26	35	40.9	34.7	38.0
Subtotal	2607	90	111	138	3.5	4.3	5.3	35	34	47	38.9	30.6	34.1
Both sexes													
30–49 years	1848	41	65	83	2.2^†^	3.5^†^	4.5	18	17	27	43.9	26.2	32.5
≥ 50 years	3269	151	168	201	4.6	5.1	6.1	61	62	78	40.4	36.9	38.8
Total	5117	192	233	284	3.8^†^	4.6^†^	5.6	79	79	105	41.1	33.9	37.0

PG, pepsinogen; Test 1, GastroPanel (Biohit HealthCare, Helsinki, Finland); Test 2, LZ-Test (Eiken Chemical Co., Ltd, Tokyo, Japan); Test 1 + 2, two tests in parallel

^*^Test 1 + 2 indicated the parallel combination of two tests (either one of the two tests was positive)

^†^*P* < 0.05 in the comparison between test 1 and test 2

Results of the PPV and detection rate are shown in Table 3. In addition to the overall results, the results were also stratified by age, sex, and test brand. The overall PPV (i.e., two-test combination) for atrophic gastritis was slightly lower than that of the individual test with either GastroPanel^®^ or LZ-Test^®^ (12.4% vs. 16.3% and 16.3%, both *P* = 0.45). Findings for intestinal metaplasia were similar (18.9% vs. 23.8% and 21.3%, *P* = 0.42 and 0.69, respectively). Taking into consideration the referral rates, the detection rate for atrophic gastritis was the same for overall and the individual tests (2.5 per 1000). The detection rate for intestinal metaplasia was also comparable (3.9 vs. 3.7 and 3.3 per 1000, *P* = 0.60 and 0.10, respectively).

**Table 3:** The performance indicators of screening, stratified by the age, sex, and the brands of tests

Brands of PG test ^†^	Positive predictive value (%)*						Detection rate (per 1000)*					
	Atrophic gastritis			Intestinal metaplasia			Atrophic gastritis			Intestinal metaplasia		
	Test 1	Test 2	Test 1 + 2	Test 1	Test 2	Test 1 + 2	Test 1	Test 2	Test 1 + 2	Test 1	Test 2	Test 1 + 2
Male												
30–49 years	10.0	10.0	6.7	10.0	11.1	6.7	1.2	1.2	1.2	1.2	1.2	1.2
≥ 50 years	20.6	19.4	16.3	32.3	28.0	25.6	4.2	4.2	4.2	6.6	6.6	6.6
Subtotal	18.2	17.4	13.8	27.3	26.7	20.7	3.2	3.2	3.2	4.8	4.8	4.8
Female												
30–49 years	11.1	12.5	8.3	11.1	11.1	7.7	1.0	1.0	1.0	1.0	1.0	1.0
≥ 50 years	14.8	15.4	11.4	22.2	15.4	20.0	2.5	2.5	2.5	3.7	2.5	4.4
Subtotal	13.8	14.7	10.6	19.4	14.3	16.7	1.9	1.9	1.9	2.7	1.9	3.1
Both sexes												
30–49 years	10.5	11.1	7.4	10.5	11.1	7.1	1.1	1.1	1.1	1.1	1.1	1.1
≥ 50 years	18.0	17.7	14.1	27.9	24.2	23.1	3.4	3.4	3.4	5.2	4.6	5.5
Total	16.3	16.3	12.4	23.8	21.3	18.9	2.5	2.5	2.5	3.7	3.3	3.9

PG = pepsinogen; Test 1 = GastroPanel (Biohit HealthCare, Helsinki, Finland); Test 2 = LZ-Test (Eiken Chemical Co., Ltd, Tokyo, Japan); Test 1 + 2 = two tests in parallel

*Positive predictive value was defined as the number of participants with premalignant lesions/ the number of participants positive to PG tests having attended an upper endoscopy. Detection rate was defined as the number of participants with premalignant lesions detected/ the number of participants having received the PG testing

^†^None reaches the significant *P* level of < 0.05 in the comparison between Test 1 and Test 2

The PPVs for either atrophic gastritis or intestinal metaplasia was slightly higher in males than females, and was significantly higher in the older-age than the younger-age group. The detection rates for atrophic gastritis and intestinal metaplasia were both higher in males than females, and were both higher in the older-age than the younger-age group.

### Comparisons of the measures between two tests

**Fig. 2:**
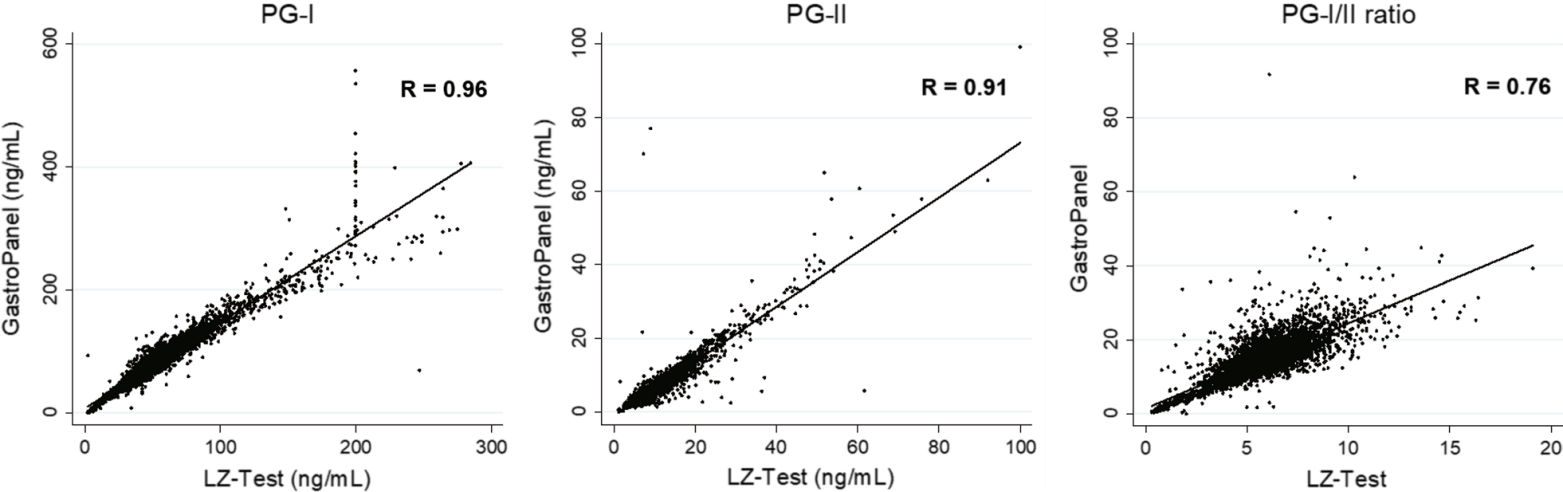
Correlations of numerical measures between two tests using scatter plots. The R indicates the correlation coefficient (Y axis: GastroPanel and X axis: LZ-Test). The linear regression lines for the PG-I, PG-II, and PG-I/II ratio are Y = 1.40X + 7.78, Y = 0.74X-0.99, and Y = 2.31X + 1.45, respectively

As shown in Fig. 2, the quantitative measures of PG-I, PG-II, and PG-I/II ratio for GastroPanel^®^ were about 40% higher, 26% lower, and 2.3-fold higher than those for LZ-Test^®^, respectively. However, the correlation coefficients between these two tests were 0.96 (95% CI 0.95–0.96), 0.91 (95% CI 0.90–0.91), and 0.76 (95% CI 0.75–0.77) for the PG-I, PG-II and the PG-I/II ratio, respectively, indicating a strong correlation (all *P* < 0.001). For the positivity rate, the kappa value for agreement was 0.65 (95% CI 0.60–0.70), also indicating a substantial agreement (*P* < 0.001).

### Comparisons of the screening indicators between two tests

In the comparison between two tests, the positivity rate was significantly lower in GastroPanel^®^ than that in LZ-Test^®^ (3.8% vs. 4.6%, *P* = 0.044), particularly in the younger-age group (2.2% vs. 3.5%, *P* = 0.018). The referral rate for diagnostic examination was slightly higher in GastroPanel^®^ than LZ-Test^®^ but the difference was not statistically significant (41.1% vs. 33.9%, *P* = 0.12). The PPVs for atrophic gastritis were the same at 16.3% (*P* = 1.00) and the results for intestinal metaplasia were 23.8% and 21.3% (*P* = 0.71) for GastroPanel^®^ and LZ-Test^®^, respectively. Two tests had the same detection rate for atrophic gastritis, 2.5 per 1000, and their detection rates as for intestinal metaplasia were also comparable (3.7 vs. 3.3 per 1000, *P* = 0.27).

### Multiple regression analyses

Results for the logistic regression analyses are shown in Table 4. Univariate analyses showed non-significant differences between the two test brands regarding the PPV for atrophic gastritis (OR 1.00, 95% CI 0.42–2.38, *P* = 1.00) or intestinal metaplasia (OR 1.16, 95% CI 0.54–2.48, *P* = 0.70). Taking into consideration the referral rate for endoscopy, the results for the detection rate were still similar between two tests for either atrophic gastritis (OR 1.00, 95% CI 0.45–2.23, *P* = 1.00) or intestinal metaplasia (OR 1.13, 95% CI 0.57–2.21, *P* = 0.73).

**Table 4:** Comparisons of the performance indicators between two brands of pepsinogen testing using the logistic regression models

Model^*^	Odds ratio	95% CI
Positive predictive value for atrophic gastritis		
Model 1		
Test 1 vs. Test 2	1.00	0.42–2.38
Model 2		
Test 1 vs. Test 2	1.12	0.40–3.12
Age ≥ 50 vs. 30–49 years	3.68	0.67–20.16
Male vs. female	1.11	0.34–3.61
Positive predictive value for intestinal metaplasia		
Model 1		
Test 1 vs. Test 2	1.16	0.54–2.48
Model 2		
Test 1 vs. Test 2	1.75	0.65–4.67
Age ≥ 50 vs. 30–49 years	5.97	1.12–31.77^†^
Male vs. female	2.84	0.94–8.55
Detection rate for atrophic gastritis		
Model 1		
Test 1 vs. Test 2	1.00	0.45–2.23
Model 2		
Test 1 vs. Test 2	1.00	0.45–2.24
Age ≥ 50 vs. 30–49 years	5.61	1.30–24.15^†^
Male vs. female	1.37	0.58–3.25
Detection rate for intestinal metaplasia		
Model 1		
Test 1 vs. Test 2	1.13	0.57–2.21
Model 2		
Test 1 vs. Test 2	1.20	0.60–2.40
Age ≥ 50 vs. 30–49 years	8.91	2.12–37.51^†^
Male vs. female	2.23	1.01–4.96^†^

CI, confidence interval; Test 1, GastroPanel (Biohit HealthCare, Helsinki, Finland); Test 2, LZ-Test (Eiken Chemical Co., Ltd, Tokyo, Japan)

^*^Model 1: the univariate logistic regression model; model 2: the multivariate logistic regression model also adjusted for body mass index, social habits, and the medical histories, in addition to the age and sex

^†^*P* < 0.05

When adjusted for age, sex, body mass index, social habits, and medical histories, the differences in PPVs for atrophic gastritis (adjusted OR 1.12, 95% CI 0.40–3.12, *P* = 0.83) (Additional file 1: Figure S1) and intestinal metaplasia (adjusted OR: 1.75, 95% CI 0.65–4.67, *P* = 0.27) (Additional file 1: Figure S2) between two tests were still non-significant. Results for the detection rates for atrophic gastritis (adjusted OR 1.00, 95% CI 0.45–2.24, *P* = 1.00) (Additional file 1: Figure S3) and intestinal metaplasia (adjusted OR 1.20, 95% CI 0.60–2.40, *P* = 0.53) (Additional file 1: Figure S4) were also non-significant.

Older age was borderline significantly associated with a higher PPV for atrophic gastritis (adjusted OR 3.68, 95% CI 0.67–20.16, *P* = 0.13) while older age was significantly associated with a higher PPV for intestinal metaplasia (adjusted OR 5.97, 95% CI 1.12–31.77, *P* = 0.036). Between sexes, differences in PPV were not statistically significant. The detection rate for atrophic gastritis was significantly higher in the older age group (adjusted OR 5.61; 95% CI 1.30–24.15, *P* = 0.021) and slightly higher for male sex (adjusted OR 1.37, 95% CI 0.58–3.25, *P* = 0.47). Regarding intestinal metaplasia, significantly higher detection rates were noted for either the older age group (adjusted OR 8.91; 95% CI 2.12–37.51, *P* = 0.003) or the male sex group (adjusted OR 2.23; 95% CI 1.01–4.96, *P* = 0.049).

### Comparison of positivity rates according to OLGA and OLGIM stages

**Fig. 3:**
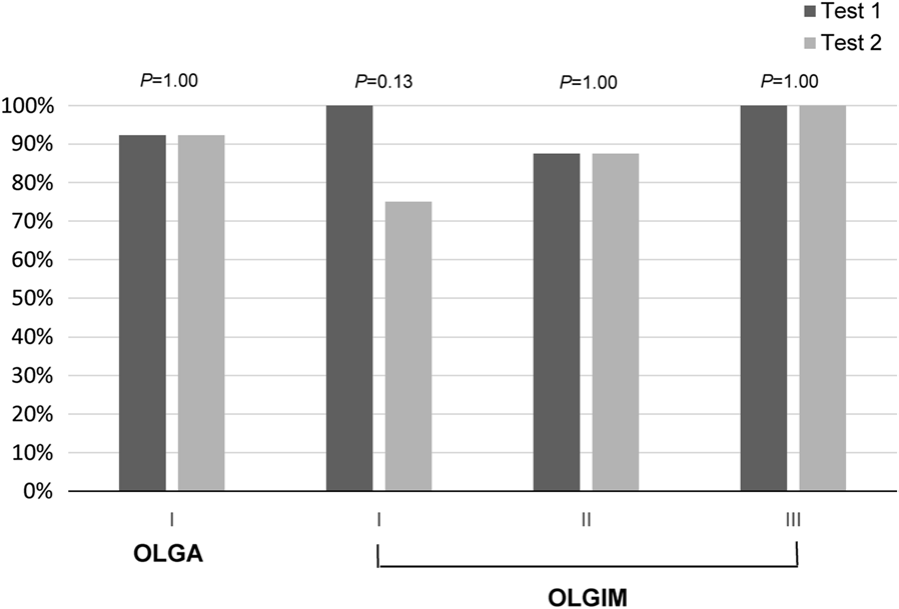
Comparisons of the positivity rates between two PG tests in patients with histologically documented premalignant lesions. Test 1 = GastroPanel (Biohit HealthCare, Helsinki, Finland) and Test 2 = LZ-Test (Eiken Chemical Co., Ltd, Tokyo, Japan)

In subjects with histologically documented atrophic gastritis and intestinal metaplasia, 84.6% and 78.9% of them tested positive for both tests, respectively. The details are shown in Fig. 3; in patients with atrophic gastritis (all were graded with OLGA stage 1 diseases), both 92.3% yielded positive results for GastroPanel^®^ and LZ-Test^®^. In patients with intestinal metaplasia, 94.7% and 84.2% yielded positive results for GastroPanel^®^ and LZ-Test^®^, respectively; the difference was not significant (*P* = 0.29). In details, the positivity rates were remarkably similar in patients with either the OLGIM stage 1 (100% vs. 75.0%, *P* = 0.13), stage 2 (87.5% vs. 87.5%, *P* = 1.00), or the stage 3 diseases (100% vs. 100%, *P* = 1.00).

## Discussion

This community-based study not only confirmed the applicability of PG testing for mass screening, but also made extensive comparisons between two different brands of PG testing. We found that although their absolute measures of PG-I, PG-II, and the PG-I/II ratio were significantly different, the correlations between them were high and the agreements in test results were substantial. We also found that, although they applied different immunoassay methods for measurement and defined different cutoff criteria, the performance of the two tests was comparable in the prediction of premalignant gastric lesions, particularly in terms of PPV and detection rate. The findings were replicated in multiple regression analysis that adjusted for differences in the baseline characteristics of participants. The histological severities of the identified atrophic gastritis and intestinal metaplasia were also similar based on unified scoring systems. Collectively, the results indicate that two different brands of PG tests perform equivalently in predicting premalignant gastric lesions in the real-world environment, supporting their interchangeability.

We evaluated the real-world performance of PG testing according to the step-by-step principles of screening [26]. First, our high population participation rate of about 67% supported PG testing as a tool for mass screening. Second, regarding the referral for endoscopic diagnosis, the endoscopic rate of about one third was relatively low, which was due to the invasive nature and the lower population acceptability of endoscopic screening. Third, regarding the PPV, the value of about one fifth has indicated the moderate predictability of PG testing in a high-risk population [27]. Finally, the detection rate of about 3 cases per 1000 participants, which took into account the positivity rate, referral rate, and the PPV, replicated the results of population-wide screening programs for other types of cancers [28].

Our study demonstrated a high correlation of quantitative measures between the two test brands, which is supported by two previous studies. First, one Japanese study, based on 304 blood samples, showed the correlation coefficients of 0.98, 0.98, and 0.92, respectively, for the PG-I, PG-II, and PG-I//II ratio between GastroPanel^®^ and LZ-Test^®^ [11]. Another Latvian study, based on 805 blood samples, also showed the high correlation coefficients of 0.89, 0.90, and 0.86 for the PG-I, PG-II, and PG-I//II ratio, respectively, between these two tests [13]. However, whether these results could be interpreted as equivalent in the real-world setting remained unclear. It was primarily because their absolute measures and cutoff definitions were so different. In our study, the prevalence rate of the premalignant conditions has declined after mass eradication of *H. pylori* [14] so the PPVs of the PG tests also decreased. As a result, the overlap of positive results between two tests may appear lower (141/284, 49.6%). Nonetheless, when we focused on the subjects with histologically documented premalignant conditions, a large proportion of them tested positive for both tests. Therefore, when we interpreted the findings with the concept of sensitivity (the number of test positives divided by the number of patients diagnosed with premalignant lesions), which was not affected by the lower prevalence rate of the premalignant lesions, the test consistency was actually high.

In our study, we applied the two tests in parallel so the prevalence rates of premalignant gastric lesions were the same. The observed similarities in the PPVs (i.e., the prevalence rate of premalignant gastric lesions multiplied by the positive likelihood ratio of the screening test) would indicate that the positive likelihood ratios of two tests should be close to each other. Using histology as the reference standard, the positive likelihood ratio (i.e., the sensitivity divided by one minus specificity) was determined with the diagnostic accuracy study. Our findings supported the robustness of cutoff criteria selection from both companies because their criteria were defined based on the judicious tradeoffs between true positive and false positive results in the receiver operating characteristic curve analyses, which had been optimized according to the similar need for clinical practice [20, 22, 29].

Our previous study has estimated a prevalence rate of about 3% for premalignant gastric lesions in this population that had undergone six rounds of mass eradication [3, 14]. Given the PPVs of about 16% observed in this study, the positive likelihood ratio of PG testing could be estimated at about 5, which was consistent with the results from the previous studies investigating subjects without *H. pylori* eradication [20, 22, 29]. Given this test capability, we also evaluated how to improve the PPV on a real-world setting. First, we applied two tests in parallel, hoping to increase the test sensitivity so as to reduce the false-negative results. However, this hypothesis was rejected as the PPV and detection rate were comparable when the tests were either applied alone or in combination. It was likely because these two tests were highly equivalent in the test capability and their results largely overlapped. Second, we performed stratified analyses according to sex and age and conducted multiple regression analyses, hoping to identify useful indicators to focus on the subgroups with the highest risk to increase the pretest possibility. We found that these tests performed more accurately in older participants. For participants aged 50 years or more, there were 3.7- and 6.0-fold increases in the PPVs for atrophic gastritis and intestinal metaplasia, and 5.6- and 8.9-fold increases in the detection rates for atrophic gastritis and intestinal metaplasia, respectively, as compared with the younger age group. These findings lend support to the recommendations of several consensus statements about the utilization of surveillance endoscopy to detect gastric cancer after *H. pylori* eradication [30, 31].

The strengths of our study include the invitation of all candidates in a high-risk community, the step-by-step evaluation following pre-established screening principles, and the head-to-head comparisons of two widely used PG tests in a real-world environment. Our findings can not only be applied in a high-risk population, but can also be generalized to other high-risk populations living in lower-risk countries, such as first-generation immigrants from high-prevalence areas. However, certain limitations should be noted. First, although our study confirmed the consistent performance of PG testing in predicting the presence of premalignant gastric lesions, detecting gastric cancer would have required a longer follow-up of this population, as our previous study has done [10]. Second, research has shown that, after *H. pylori* eradication, the histology can be improved but genetic damage may persist, which would lead to a decreased capacity of PG testing in the prediction of gastric cancer risk [17, 32]. Our study demonstrated a low prevalence rate of advanced-stage pre-malignancies (i.e., stage III–IV OLGA and OLGIM), particularly atrophic gastritis, which was likely related to the effect of *H. pylori* eradication on gastritis regression [14, 17]. Other serological markers or molecular testing based on histological samples could be possible solutions and directions for future research [33, 34]. Finally, regarding the generalizability, although we have demonstrated the equivalent performance between these two brands of PG tests for mass screening, it still need further investigate the interchangeability for other methods to measure the PG concentration except ELISA and L-TIA.

## Conclusions

We demonstrate high correlations of either the PG-I, PG-II, or PG-I/II measures, good agreements in the test positivity, and the equivalent performance between two brands of PG tests for mass screening, which support the interchangeability of their test results. We also show that targeting older participants will increase the predictability of PG testing in a high-risk population when *H. pylori* screening and treatment has been implemented as common practice. These findings provide important implications in identifying those who will retain gastric cancer risk to properly identify who will benefit most from endoscopic screening.

## Supplementary Information

**Additional file 1.** The baseline data of endoscopic receivers/refusers, and the multivariate logistic regression models between the two pepsinogen test brands.

## Data Availability

Most data relevant to the study are included in the article. Additional data are available by contacting the corresponding author.

## Abbreviations

PG: Pepsinogen
CI: Confidence interval
*H. pylori*: *Helicobacter pylori*
OLGA: Operative Link for Gastritis Assessment of Atrophic Gastritis
OLGIM: Operative Link for Gastritis Assessment of Intestinal Metaplasia
OR: Odds ratio
PPV: Positive predictive value
