# Supplementary material for: Brand interchangeability of pepsinogen tests in the real-world setting after eradication of Helicobacter pylori: a community-based study
Source: BMC Gastroenterol. 2022 Feb 18;22:69. doi: 10.1186/s12876-022-02155-7 (PMC8857789; doi:10.1186/s12876-022-02155-7)
Supplement: Supplementary file 1 — Additional file 1. The baseline data of endoscopic receivers/refusers, and the multivariate logistic regression models between the two pepsinogen test brands. [file 12876_2022_2155_MOESM1_ESM.docx]

**Appendix**

This appendix has been provided by the authors to give readers additional information about their work.

Supplement to: Chiang TH, Chen YN, Chen YR, et al. Brand interchangeability of pepsinogen tests in the real-world setting after eradication of *Helicobacter pylori*: A community-based study

| Item | Description |
| --- | --- |
| Supplementary Table 1 | The comparison of baseline data between endoscopic receivers and refusers. |
| Supplementary Figure 1 | Multivariate logistic regression model between the two test brands regarding the PPV for atrophic gastritis. |
| Supplementary Figure 2 | Multivariate logistic regression model between the two test brands regarding the PPV for intestinal metaplasia. |
| Supplementary Figure 3 | Multivariate logistic regression model between the two test brands regarding the detection rate for atrophic gastritis. |
| Supplementary Figure 4 | Multivariate logistic regression model between the two test brands regarding the detection rate for intestinal metaplasia. |

**Supplementary Table 1.** The comparison of baseline data between endoscopic receivers and refusers

| **Participants** **with positive test results** | **Endoscopic receivers**  **(*n*=105)** | **Endoscopic refusers**  **(*n*=179)** | ***P* value*** |
| --- | --- | --- | --- |
| Mean age, years (SD; range) | 57.5 (11.9; 30-88) | 58.5 (14.2; 30-96) | 0.59 |
| Male sex, no. (%) | 58 (55.2) | 88 (49.2) | 0.33 |
| Body mass index, kg/m^2^ (SD; range) | 25.6 (4.0; 19.3-52.1) | 24.8 (3.9; 15.8-37.2) | 0.08 |
| Social habits, no. (%) |  |  |  |
| Current smoker | 20 (19.0) | 24 (13.4) | 0.21 |
| Regular alcohol drinking | 4 (3.8) | 9 (5.0) | 0.64 |
| Betel nut chewing | 4 (3.8) | 9 (5.0) | 0.64 |
| Medical history, no. (%) |  |  |  |
| Hypertension | 39 (37.1) | 71 (40.0) | 0.63 |
| Diabetes mellitus | 8 (7.6) | 15 (8.4) | 0.27 |
| Hyperlipidemia | 14 (13.3) | 15 (8.4) | <0.001* |
| Cardiovascular disease | 8 (7.6) | 8 (4.5) | 0.007* |
| Stroke | 1 (1.0) | 0 (0) | 0.18 |
| Chronic hepatitis B | 17 (16.2) | 23 (12.8) | 0.90 |
| Chronic hepatitis C | 1 (1.0) | 2 (1.1) | 0.94 |
| Chronic kidney disease | 1 (1.0) | 1 (0.6) | 0.71 |

Abbreviation: SD=standard deviation

**P* <0.05 in the comparison between the endoscopic receivers and refusers.

**Supplementary Figure 1.** Multivariate logistic regression model between the two test brands regarding the PPV for atrophic gastritis. Test 1=GastroPanel (Biohit HealthCare, Helsinki, Finland) and Test 2=LZ-Test (Eiken Chemical Co., Ltd, Tokyo, Japan).


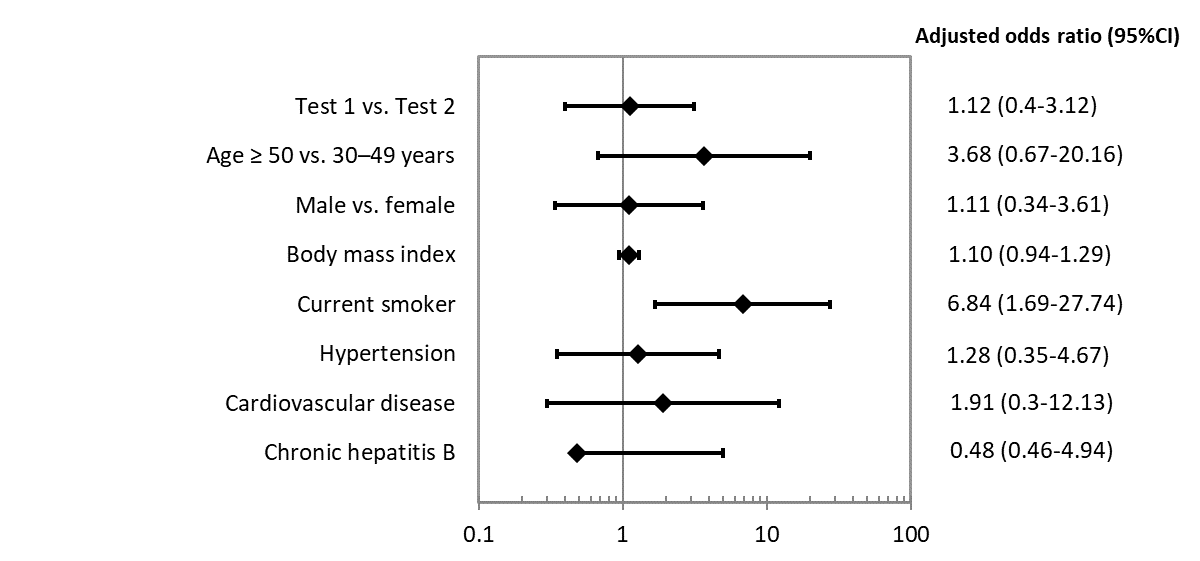


**Supplementary Figure 2.** Multivariate logistic regression model between the two test brands regarding the PPV for intestinal metaplasia. Test 1=GastroPanel (Biohit HealthCare, Helsinki, Finland) and Test 2=LZ-Test (Eiken Chemical Co., Ltd, Tokyo, Japan).

**Supplementary Figure 3.** Multivariate logistic regression model between the two test brands regarding the detection rate for atrophic gastritis. Test 1=GastroPanel (Biohit HealthCare, Helsinki, Finland) and Test 2=LZ-Test (Eiken Chemical Co., Ltd, Tokyo, Japan).

**Supplementary Figure 4.** Multivariate logistic regression model between the two test brands regarding the detection rate for intestinal metaplasia. Test 1=GastroPanel (Biohit HealthCare, Helsinki, Finland) and Test 2=LZ-Test (Eiken Chemical Co., Ltd, Tokyo, Japan).
